# Fruit and vegetable consumption and injurious falls among adults aged ≥ 50 years from low- and middle-income countries

**DOI:** 10.1007/s40520-025-02966-0

**Published:** 2025-03-17

**Authors:** Lee Smith, Guillermo F. López Sánchez, Nicola Veronese, Mark A Tully, Damiano Pizzol, Laurie Butler, Masoud Rahmati, José Francisco López-Gil, Yvonne Barnett, Louis Jacob, Pinar Soysal, Alberto Castagna, Jae Il Shin, Ai Koyanagi

**Affiliations:** 1 Centre for Health Performance and Wellbeing, Anglia Ruskin University, Cambridge, UK; 2 Division of Preventive Medicine and Public Health, Department of Public Health Sciences, School of Medicine, University of Murcia, Murcia, Spain; 3 Saint Camillus International University of Health Sciences, Rome, Italy; 4 School of Medicine, Ulster University, Londonderry, UK; 5 Italian Agency for Development Cooperation, Khartoum, Sudan; 6 CEReSS-Health Service Research and Quality of Life Center, Aix-Marseille University, Marseille, France; 7 Department of Physical Education and Sport Sciences, Faculty of Literature and Human Sciences, Lorestan University, Khoramabad, Iran; 8 Department of Physical Education and Sport Sciences, Faculty of Literature and Humanities, Vali-E-Asr University of Rafsanjan, Rafsanjan, Iran; 9 One Health Research Group, Universidad de Las Américas, Quito, 170124 Ecuador; 10 Research and Development Unit, Parc Sanitari Sant Joan de Déu, Dr. Antoni Pujadas, Sant Boi de Llobregat, Barcelona, Spain; 11 Department of Physical Medicine and Rehabilitation, Lariboisière-Fernand Widal Hospital, Assistance Publique - Hôpitaux de Paris (AP-HP), Université Paris Cité, Paris, France; 12 Epidemiology of Ageing and Neurodegenerative Diseases (EpiAgeing), U1153, Inserm, Université Paris Cité, Paris, France; 13 Department of Geriatric Medicine, Faculty of Medicine, Bezmialem Vakif University, Istanbul, Turkey; 14 Distretto di Soverato, SOC Cure Primarie, Azienda Sanitaria Provinciale di Catanzaro, Catanzaro, Italy; 15 Department of Pediatrics, Yonsei University College of Medicine, Seoul, Republic of Korea; 16 Severance Underwood Meta-Research Center, Institute of Convergence Science, Yonsei University, Seoul, Republic of Korea

**Keywords:** Adults, Low- and middle-income countries, Fruit and vegetable consumption, Falls

## Abstract

**Objective:**

Inadequate fruit and vegetable consumption may increase risk for falls. However, to date, only one study has examined this association in a sample restricted to females, while the mediators of this association are largely unknown. Therefore, we aimed to examine the association between fruit and vegetable consumption and injurious falls, and to identify potential mediators in a sample including both males and females.

**Methods:**

Cross-sectional, nationally representative data from the World Health Organization (WHO) Study on global AGEing and adult health (SAGE) were analyzed. Fruit/vegetable consumption was divided into two groups: ≥2 servings of fruits and ≥3 servings of vegetables per day (adequate consumption) or else (inadequate consumption). Fall-related injury referred to those that occurred in the past 12 months. Multivariable logistic regression and mediation analysis were conducted.

**Results:**

Data on 34,129 individuals aged ≥ 50 years were analyzed (mean age 62.4 years; 52.1% females). Overall, inadequate fruit/vegetable intake was associated with a significant 1.41 (95%CI = 1.05–1.90) times higher odds for injurious falls. This association was only significant among females (OR = 1.96; 95%CI = 1.32–2.85). Mediation analysis showed that affect (mediated percentage 8.8%), cognition (7.2%), and sleep/energy (7.5%) were significant mediators, but vision, grip strength, and gait speed were not.

**Conclusions:**

Inadequate fruit and vegetable consumption was associated with higher odds for injurious falls among adults aged ≥ 50 years (especially females), and this association was partly mediated by cognition, affect, and sleep/energy. Future longitudinal studies are necessary to provide more insight into the underlying mechanisms, and to assess whether increasing fruit/vegetable consumption may reduce risk for falls.

**Supplementary Information:**

The online version contains supplementary material available at 10.1007/s40520-025-02966-0.

## Introduction

Every year, one-third of community-dwelling older adults fall, while approximately 10–15% subsequently endure an injury [1–3]. Importantly, in 2010, falls were responsible for approximately 80% of disability from unintentional injuries excluding traffic accidents in adults aged 50 years and over [4]. Falls are the leading cause of injury mortality among older adults [5]. The burden of injurious falls is likely to increase as the global population ages, and this is particularly true for those in low- and middle-income countries (LMICs) where the United Nation estimates that two-thirds of the world’s population aged 60 years and over will live by 2050 [6]. Moreover, injurious falls may be more problematic in LMICs in relation to their management, as compared to high-income countries: LMICs are more likely to have under-resourced healthcare compounded by a fragmented healthcare system. It is clear that effective interventions are needed to reduce falls, and importantly injurious falls, among older adults in LMICs. In order to develop targeted interventions, risk factors for falls or injurious falls need to be identified in these countries.

While several risk factors for falls (e.g., impaired balance and gait, polypharmacy, visual impairment, cognitive decline, food insecurity) have been identified to date [7–12], one potentially important but understudied risk factor is that of fruit and vegetable consumption. Inadequate fruit and vegetable consumption can theoretically lead to higher risk for falls via factors such as mental health problems, cognitive impairment, visual problems, sleep problems, and low physical function and performance [13, 14]. For example, inadequate fruits and vegetables intake may affect mental health through folate deficiency, which plays a critical role in mood regulation [15], while problems with affect can increase risk for falls via impairment of gait and balance (an association that is mediated through cognitive, sensory, and motor pathways) [16]. Next, the antioxidant properties of fruits and vegetables may explain the higher risk for cognitive decline observed in people with inadequate fruit/vegetable consumption [17, 18], while cognitive impairment may increase risk of falls via problems with gait control [19]. Furthermore, fruit/vegetable consumption may protect against visual difficulties by, for example, protecting the retina from oxidative stress in the case of age-related macular degeneration [20]. Problems with navigating environmental hazards in visual impairment may lead to higher risk for falls [21]. In addition, sleep problems can be caused by inadequate fruit/vegetable intake through its impact on tryptophan availability and the synthesis of serotonin and melatonin [22]. Sleep problems, in turn, can increase risk for falls via, for instance, excessive daytime sleepiness increasing one’s risk of tripping [23]. Finally, low serum carotenoids and an increase in general frailty in inadequate fruit/vegetable consumption may be implicated in its link with poor physical performance and function [24], which can cause falls via factors such as impaired balance [25].

Despite this, to the authors’ knowledge, to date, just one study has investigated the association between fruit and vegetable consumption and falls. The study examined the prospective association of fruit/vegetable intake with fall-related hospitalizations among older women (*n* = 1429, age ≥ 70 years) from Western Australia with 14.5 years of follow-up. It was observed that fall-related hospitalizations were lower in participants consuming more vegetables (hazard ratio (HR) per 75 g serve: 0.90 (95% CI = 0.82–0.99)), but not fruit intake (per 150 g serve: 1.03 (95%CI = 0.93–1.14)). Furthermore, only total cruciferous vegetable intake was inversely associated with fall-related hospitalization (HR: per 20 g serve: 0.90 (95%CI = 0.83–0.97)) [26]. Clearly, more research is needed in other samples and settings, as well as in males and females, to gain a greater understanding of the relationship between fruit and vegetable consumption and falls, as well as to confirm or refute the findings from the only study on this topic. In particular, it is important to investigate sex-specific associations between fruit and vegetable consumption and injurious falls as previous research has found that males and females have different fall risk profiles and suggests that these differences should be considered when developing preventive strategies [27]. Moreover, some variation in adequate fruit and vegetable intake between males and females has been observed in LMICs, with males consuming less than females [28].

Given this background, the aim of the present study was to examine the association between fruit and vegetable consumption and injurious falls in a sample of 34129 individuals aged ≥ 50 years from six LMICs. A further aim was to examine to what extent affect, cognition, vision, sleep/energy, and markers of physical strength and performance (handgrip strength, gait speed) mediate the potential fruit/vegetable – injurious falls relationship.

## Materials and methods

We analyzed data from the Study on Global Ageing and Adult Health (SAGE), which was a survey undertaken in China, Ghana, India, Mexico, Russia, and South Africa between 2007 and 2010. Based on the World Bank classification at the time of the survey, all these countries were LMICs. Details of the survey methodology can be found elsewhere [29]. Briefly, in order to obtain nationally representative samples, a multistage clustered sampling design method was employed. The sample consisted of adults aged ≥ 18 years with oversampling of those aged ≥ 50 years. Trained interviewers conducted face-to-face interviews using a standard questionnaire. Standard translation procedures were undertaken to ensure comparability between countries. The survey response rates were: China 93%; Ghana 81%; India 68%; Mexico 53%; Russia 83%; and South Africa 75%. Sampling weights were constructed to adjust for the population structure as reported by the United Nations Statistical Division. Ethical approval was obtained from the World Health Organization (WHO) Ethical Review Committee and local ethics research review boards. Written informed consent was obtained from all participants.

### Fall-related injury

The variable on fall-related injury of the SAGE was derived from questions of the WHO guidelines on injuries [30]. First, the participant was asked “In the past 12 months, have you had any other event (other than a road traffic accident) where you suffered from bodily injury?” Those who answered affirmatively were prompted to the next question “What was the cause of the injury?” If there were multiple injuries, the respondent was instructed to refer to the most recent injury. If the respondent answered “Fall”, then he or she was considered to have had a fall-related injury in the past year.

### Fruit and vegetable consumption

Participants were asked the two following questions: “How many servings of fruit do you eat on a typical day?” and “How many servings of vegetables do you eat on a typical day?” Those who consumed at least two servings of fruits and three servings of vegetables were considered to have adequate fruit/vegetable consumption based on findings from a recent study that this combination of fruits and vegetables was associated with the lowest risk for mortality [31].

### Mediators

The potential mediators (i.e., affect, cognition, vision, sleep/energy, handgrip strength, gait speed) considered in this study were selected based on previous literature showing that these could be the consequence of fruit/vegetable consumption, while they can also be the cause of falls [7, 26, 32–34]. Affect, cognition, vision, and sleep/energy were assessed with two questions each that assessed health function in the past 30 days. The actual questions can be found in the Appendix. Each item was scored on a five-point scale ranging from ‘none’ to ‘extreme/cannot do’. For each separate domain, we used factor analysis with polychoric correlations to obtain a factor score which was later converted to scores ranging from 0 to 100 [35–37], with higher values representing worse health function. Weak handgrip strength was defined as < 27 kg for men and < 16 kg for women using the average value of the two handgrip measurements of the dominant hand [38]. Gait speed was based on a 4 m timed walk and was measured by asking the participant to walk at a normal pace. The interviewer recorded the time to completion of the 4 m walk. Slow gait speed referred to ≤ 0.8 m/s [38].

### Control variables

The control variables of this study were selected based on past literature [26], and included age, sex, wealth quintiles based on income, highest level of education achieved (≤ primary, secondary, tertiary), setting (rural, urban), body mass index (BMI), diabetes based on self-reported diagnosis, physical activity, smoking (never, current, past), and alcohol consumption. BMI was based on measured weight and height and was categorized as < 18.5, 18.5–24.9, 25.0-29.9, and ≥ 30 kg/m^2^. Levels of physical activity were assessed with the Global Physical Activity Questionnaire and were classified as low, moderate, and high based on conventional cut-offs [39]. Consumers of at least four (females) or five drinks (males) of any alcoholic beverage per day on at least one day in the past week were considered ‘heavy’ drinkers. Those who had ever consumed alcohol but were not heavy drinkers were categorized as ‘non-heavy’ drinkers [40].

### Statistical analysis

The statistical analysis was undertaken using Stata v14.2 (Stata Corp LP, College station, Texas). The analysis was restricted to those aged ≥ 50 years. The difference in sample characteristics by fruit/vegetable consumption status was tested by Chi-squared tests and Student’s t-tests for categorical and continuous variables, respectively. Multivariable logistic regression analysis was conducted to assess the association between inadequate fruit/vegetable consumption (exposure) and injurious falls (outcome). The analysis was done using the overall sample and also sex-stratified samples. Models adjusted only for country, and fully-adjusted models are presented. Next, mediation analysis was also conducted to gain an understanding of the extent to which affect, cognition, vision, sleep/energy, grip strength, and gait speed may explain the association between inadequate fruit/vegetable consumption and injurious falls using the overall sample. We used the khb (Karlson Holm Breen) command in Stata [41] for the mediation analysis. This method can be applied in logistic regression models and decomposes the total effect (i.e., unadjusted for the mediator) of a variable into direct and indirect effects. Using this method, the percentage of the main association explained by the mediator can also be calculated (mediated percentage). Each potential mediator was included in the model individually.

All regression analyses including the mediation analysis were adjusted for age, sex, wealth, education, setting, BMI, diabetes, physical activity, smoking, alcohol consumption, and country, with the exception of the sex-stratified analysis which was not adjusted for sex. Adjustment for country was done by including dummy variables for each country in the model as in previous SAGE publications [42, 43]. Under 5% of the data were missing for all the variables included in the analysis with the exception of fruit/vegetable (7.6%), BMI (6.2%), grip strength (10.6%), and gait speed (9.8%). Complete case analysis was done. The sample weighting and the complex study design were taken into account in all analyses. Results from the regression analyses are presented as odds ratios (ORs) with 95% confidence intervals (CIs). The level of statistical significance was set at *P* < 0.05.

## Results

**Fig. 1:**
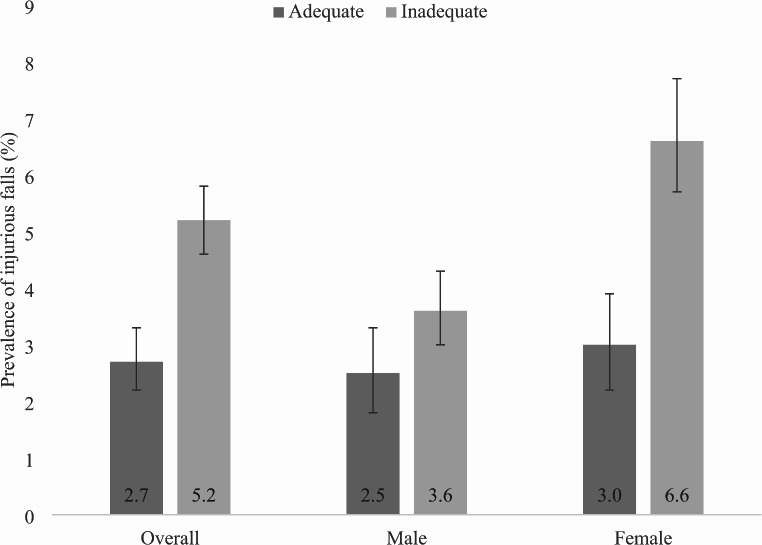
Prevalence of injurious falls by adequate or inadequate fruit/vegetable intake in the overall and sex-stratified samples Bars denote 95% confidence interval

Data on 34129 individuals aged ≥ 50 years were analyzed. The sample size of each country was: China *n* = 13,175; Ghana *n* = 4305; India *n* = 6560, Mexico *n* = 2313; Russia *n* = 3938; South Arica *n* = 3838. The prevalence of inadequate fruit/vegetable intake and injurious falls were 67.2% and 4.2%, respectively. The sample characteristics are shown in Table 1. The mean age was 62.4 years and 52.1% were females. Those with inadequate fruit/vegetable intake were significantly more likely to be poorer, with lower levels of education, and be from rural settings. Furthermore, they were also significantly more likely to be underweight, have high levels of physical activity, be smokers, and have weak handgrip strength and slow gait, while they also had significantly worse health status scores for affect, cognition, vision, and sleep/energy. The prevalence of injurious falls was higher among people with inadequate fruit/vegetable intake especially among females (Fig. 1). After adjustment for potential confounders, in the overall sample, inadequate fruit/vegetable intake was associated with a significant 1.41 (95%CI = 1.05–1.90) times higher odds for injurious falls (Table 2). This association was particularly pronounced among females (OR = 1.96; 95%CI = 1.32–2.85) and was not significant among males. Mediation analysis showed that affect (mediated percentage 8.8%), cognition (7.2%), and sleep/energy (7.5%) were significant mediators in the association between inadequate fruit/vegetable intake and injurious falls, but vision, grip strength, and gait speed were not significant mediators (Table 3).

**Table 1:** Sample characteristic (overall and by fruit/vegetable consumption status)

			Fruit/vegetable consumption		
Characteristic		Overall	Adequate (32.8%; *n* = 10336)	Inadequate (67.2%; *n* = 21214)	P-value^a^
Age	Mean (SE)	62.4 (0.2)	62.1 (0.3)	62.4 (0.2)	0.260
Sex	Female	52.1	52.4	51.9	0.554
	Male	47.9	47.6	48.1	
Wealth	Poorest	17.1	9.7	20.1	< 0.001
	Poorer	19	15.8	20.3	
	Middle	19.5	20.9	18.8	
	Richer	21.3	25.1	19.9	
	Richest	23.1	28.5	20.9	
Education	≤Primary	57.4	50.1	63.5	< 0.001
	Secondary	35.2	42	30.2	
	Tertiary	7.4	7.9	6.3	
Setting	Rural	53.8	39.3	61.4	< 0.001
	Urban	46.2	60.7	38.6	
BMI (kg/m^2^)	< 18.5	16.7	4.8	23.3	< 0.001
	18.5–24.9	47.6	52.9	45.8	
	25.0-29.9	24.2	32.1	19.7	
	≥ 30.0	11.5	10.2	11.2	
Diabetes	No	93.2	93.3	93.1	0.718
	Yes	6.8	6.7	6.9	
Physical activity	High	49.1	44.4	51.6	< 0.001
	Moderate	22.8	28.3	20.9	
	Low	28.1	27.3	27.5	
Smoking	Never	58.6	67.9	53.5	< 0.001
	Current	34.9	25.1	40.1	
	Past	6.6	6.9	6.5	
Alcohol consumption	Never	67.1	68.4	68	0.023
	Non-heavy	28.8	26.9	28.5	
	Heavy	4.1	4.8	3.5	
Affect^b^	Mean (SE)	21.1 (0.5)	10.6 (0.4)	26.1 (0.6)	< 0.001
Cognition^b^	Mean (SE)	30.6 (0.5)	24.0 (0.6)	33.9 (0.7)	< 0.001
Vision^b^	Mean (SE)	28.8 (0.4)	23.5 (0.5)	31.6 (0.6)	< 0.001
Sleep/energy^b^	Mean (SE)	27.4 (0.5)	19.1 (0.5)	31.1 (0.6)	< 0.001
Weak handgrip strength	No	66.8	70.5	64.3	0.004
	Yes	33.2	29.5	35.7	
Slow gait speed	No	63.2	77.1	57.1	< 0.001
	Yes	36.8	22.9	42.9	

Abbreviation: SE Standard error; BMI Body mass index

Data are % unless otherwise stated

^a^ P-value was based on Chi-squared tests for categorical variables and Student’s t-tests for continuous variables

^b^ Scores ranged from 0 to 100 with higher scores corresponding to worse health status

**Table 2:** Association between inadequate fruit/vegetable consumption and injurious falls (outcome) estimated by multivariable logistic regression (overall sample and samples stratified by sex)

	Models adjusted only for country			Fully adjusted models		
Sample	OR	95%CI	P-value	OR	95%CI	P-value
Overall	1.51	[1.15,1.97]	0.003	1.41	[1.05,1.90]	0.023
Only males	1.13	[0.75,1.72]	0.551	0.95	[0.62,1.43]	0.793
Only females	1.87	[1.38,2.54]	< 0.001	1.96	[1.35,2.85]	< 0.001

Abbreviation: OR Odds ratio; CI Confidence interval

Fully adjusted models are adjusted for age, sex, wealth, education, setting, body mass index, diabetes, physical activity, smoking, alcohol consumption, and country, except for the sex-stratified samples which were not adjusted for sex

**Table 3:** Mediators in the association between inadequate fruit/vegetable consumption and injurious falls (outcome)

Mediator	Effect	OR [95%CI]	*P*-value	% Mediated^a^
Affect	Total	1.39 [1.03,1.87]	0.031	8.8
	Direct	1.35 [1.00,1.81]	0.048	
	Indirect	1.03 [1.01,1.05]	0.008	
Cognition	Total	1.41 [1.05,1.89]	0.023	7.2
	Direct	1.37 [1.02,1.84]	0.034	
	Indirect	1.02 [1.01,1.04]	0.004	
Vision	Total	1.41 [1.05,1.89]	0.023	NA
	Direct	1.40 [1.04,1.88]	0.025	
	Indirect	1.01 [1.00,1.02]	0.294	
Sleep/energy	Total	1.41 [1.05,1.89]	0.024	7.5
	Direct	1.37 [1.02,1.84]	0.036	
	Indirect	1.03 [1.01,1.05]	0.009	
Grip strength	Total	1.39 [1.02,1.89]	0.038	NA
	Direct	1.38 [1.01,1.88]	0.042	
	Indirect	1.01 [1.00,1.02]	0.109	
Gait speed	Total	1.37 [1.02,1.84]	0.039	NA
	Direct	1.36 [1.01,1.83]	0.043	
	Indirect	1.01 [1.00,1.01]	0.178	

Abbreviation: OR Odds ratio; CI Confidence interval

Models are adjusted for age, sex, wealth, education, setting, body mass index, diabetes, physical activity, smoking, alcohol consumption, and country

^a^ The mediated percentage was only calculated in the presence of a significant indirect effect (*P* < 0.05)

## Discussion

### Main findings

In the present study including large representative samples of middle-aged to older adults from six LMICs, it was observed that inadequate fruit/vegetable intake was associated with a significant 1.41 (95%CI = 1.05–1.90) times higher odds for injurious falls, with the association being significant only among females (OR = 1.96; 95%CI = 1.35–2.85). Interestingly, the mediation analysis showed that only cognition, affect and sleep/energy were significant mediating variables (although it is important to highlight that mediating percentages were modest), while vision, grip strength, and gait speed were not. A visual display of the potential mediating effect is shown in Appendix 1. To the best of our knowledge, this is the first study on this topic to include both females and males, while it is also the first to quantify the degree to which several potential mediators may explain the association between inadequate fruit/vegetable consumption and (injurious) falls.

### Interpretation of the findings

The findings from the present study are broadly in line with the only previous study on this topic (restricted to females) which found that vegetable intake is associated with lower risk for falls, prospectively [26]. Our study adds to this previous literature by showing for the first time that inadequate vegetable/fruit intake is associated with increased odds for injurious falls only among females and not males, and that the association is partly mediated by cognition, affect, and sleep/energy. There are several plausible mechanisms that may explain the fruit/vegetable – injurious falls relationship. First, the identified potential mediators may be implicated in the causal pathway. As previously discussed, inadequate fruit/vegetable consumption is associated with cognitive impairment, possibly owing to fruit and vegetables having a high antioxidant content, which protects against oxidative damage to the brain. In turn, cognitive impairment likely increases risk of falls via the role cognition plays in balance. Indeed, all aspects of balance control deteriorate with increasing severity of cognitive impairment, and executive function plays an important role in balance control [44]. Next, impairment in the synthesis of serotonin and melatonin may be implicated in the increased odds for sleep problems among people with inadequate fruit/vegetable consumption. Tryptophan is found in abundance in fruit and vegetables [45], and is a precursor of the neurotransmitter serotonin and the neurosecretory hormone melatonin, both of which are linked to sleep and alertness [22]. Importantly, sleep problems likely result in a higher odd of injurious falls owing to excessive daytime sleepiness which contributes to impairments in behavioral and cognitive functions similarly implicated in falls as stated above [46]. Finally, affect was also identified as a potential mediator. Fruit and vegetable consumption is associated with depression likely owing to folate that plays a critical role in mood regulation, as well as their anti-inflammatory properties such as polyphenols (e.g., present in blueberries), and their abundance of free radicals that can reduce oxidative stress [15, 47]. In turn, depression may increase fall risk via impairment in balance, that is often mediated through cognitive, sensory, and motor pathways [16]. However, it is important to note that these three mediating variables only explained a small proportion of the variation, and future research is now needed to explore other potential pathways.

The finding that a significant association between fruit and vegetable consumption and injurious falls was found only among females is interesting. Such an association is difficult to explain but it may be that, for example, fruit/vegetable intake has a stronger effect on the potential mediators among females than in males. For instance, in a study including 1165 adults who were low consumers of fruit and vegetables (< 3 servings/day) at baseline, females who increased fruit and vegetable intake by ≥ 3 servings showed improvements in insomnia symptoms, sleep quality, and time to fall asleep compared to women who did not change or decreased their fruit and vegetable intake at 3-month follow-up, but associations were not as apparent among men [48]. This could be explained, for example, by magnesium intake, a nutrient found in leafy green vegetables, which has been associated with improvements in insomnia symptoms [49], and for which daily requirements differ by gender [50].

### Implication of study findings and areas for future research

Findings from the present study suggest that promotion of ≥ 2 servings of fruits and ≥ 3 serving of vegetables per day, which has been associated with the lowest mortality risk [31], may also help reduce risk for injurious falls especially among women. Importantly, several global plans have been developed to increase fruit and vegetable intake; these plans include WHO Global Action Plan for the Prevention and Control of NCDs, and the United Nations (UN) Decade of Action on Nutrition 2016–2025. While these initiatives are mainly for the prevention of non-communicable diseases such as cardiovascular disease and cancer, our study results show that these may also help prevent injurious falls among women. However, future longitudinal and intervention studies are necessary to assess temporal associations, as well as whether increasing fruit/vegetable intake can reduce risk for falls. Furthermore, future research should assess whether the sex-differences observed in our study can be replicated, and if so, the mechanisms underlying this difference should be investigated.

### Strengths and limitations

The large representative samples of middle-aged and older adults across six LMICs, and the use of mediation analysis to quantify the extent to which potential mediators may explain the fruit/vegetable intake-injurious fall relationship are clear strengths of the present study. However, findings must be interpreted in light of the study limitations. First, the study was cross-sectional in nature, and thus, the direction of the association is not known. It is plausible that injurious falls lead to a reduction in fruit and vegetable consumption, for example, by hindering one’s ability to cook or shifting cooking responsibilities from one person to another, but future work of a qualitative nature is required to test this hypothesis. Relatedly, given the cross-sectional design of the study, and the different ways in which fruit/vegetable consumption, injurious falls, and the potential mediators can be intertwined, it is possible for the mediated percentages calculated in our study to be an overestimation. Future studies of longitudinal design would be able to provide more insight into the potential mechanisms. Second, the majority of questions were self-reported potentially introducing recall and social desirability bias into the findings. Third, we also lacked information on the exact type of fruit or vegetable people consume, despite the fact that some types of vegetables or fruits may be more beneficial for fall prevention. We were also unable to conduct country-specific analysis as meaningful results could not be obtained due to the limited sample size. Future multi-country studies with larger sample size and more detailed information on the specific type of fruit/vegetable consumed can potentially shed light on whether the association between fruit/vegetable consumption and injurious falls differs by country and if so whether the difference is attributable to the different types of fruit/vegetables consumed. Finally, it is also possible that people who consume greater amounts of fruits and vegetables are those who are more health-conscious and consume other types of food (such as oily fish and dairy products) that may aid in the prevention of falls.

## Conclusions

In the present study including large representative samples of middle-aged to older adults from six LMICs, inadequate fruit and vegetable consumption was associated with higher odds for injurious falls, and this association was partly mediated by cognition, affect, and sleep/energy. Interventions to increase fruit and vegetable consumption may also have the additional benefit to prevent injurious falls, especially among females, as well as a plethora of non-communicable diseases, pending future longitudinal research. However, it is important to note that diets high in fruit and vegetables are often not affordable to those residing in LMICs, and thus, any intervention will require strong governmental commitment to address economic challenges in this context.

## Electronic supplementary material

Below is the link to the electronic supplementary material.

Supplementary Material 1

## Data Availability

Data supporting the findings of this study are available from the corresponding authors upon reasonable request.

## Appendix

**Table:**

Questions used to assess cognition, affect, vision, and sleep and energy	
**Cognition**	(1) Overall in the last 30 days, how much difficulty did you have with concentrating or remembering things?
	(2) Overall in the last 30 days, how much difficulty did you have in learning a new task (for example, learning how to get to a new place, learning a new game, learning a new recipe)?
**Affect**	(1) Overall in the last 30 days, how much of a problem did you have with feeling sad, low or depressed?
	(2) Overall in the last 30 days, how much of a problem did you have with worry or anxiety?
**Vision**	(1) In the last 30 days, how much difficulty did you have in seeing and recognizing an object or a person you know across the road (i.e. from a distance of about 20 m)?
	(2) In the last 30 days, how much difficulty did you have in seeing and recognizing an object at arm’s length (for example, reading)?
**Sleep and energy**	(1) Overall in the last 30 days, how much of a problem did you have with sleeping, such as falling asleep, waking up frequently during the night or waking up too early in the morning?
	(2) Overall in the last 30 days, how much of a problem did you have due to not feeling rested and refreshed during the day (e.g. feeling tired, not having energy)?
