# Supplementary material for: Fruit and vegetable consumption and injurious falls among adults aged ≥ 50 years from low- and middle-income countries
Source: Aging Clin Exp Res. 2025 Mar 17;37(1):90. doi: 10.1007/s40520-025-02966-0 (PMC11913954; doi:10.1007/s40520-025-02966-0)
Supplement: Supplementary file 1 — Supplementary Material 1 [file 40520_2025_2966_MOESM1_ESM.docx]

**Appendix 1**

Impaired cognition

Injurious falls

Inadequate fruit/vegetable intake

Sleep problems

Affective problems
